# When Facebook Becomes a Part of the Self: How Do Motives for Using Facebook Influence Privacy Management?

**DOI:** 10.3389/fpsyg.2021.769075

**Published:** 2021-12-16

**Authors:** Hyunjin Kang, Wonsun Shin

**Affiliations:** ^1^Wee Kim Wee School of Communication and Information, College of Humanities, Arts, and Social Sciences, Nanyang Technological University, Singapore, Singapore; ^2^Media and Communications, School of Culture and Communication, The University of Melbourne, Parkville, VIC, Australia

**Keywords:** social networking sites, SNSs, Facebook, online privacy management, digital self-extension

## Abstract

This study examines how three different motivations for using an SNS (i.e., self-expression, belonging, and memory archiving) influence multi-facets of privacy boundary management on the platform mediated by self-extension to it. In recognition of the fact that information management on SNSs often goes beyond the “disclosure-withdrawal” dichotomy, the study investigates the relationships between the three SNS motives and privacy boundary management strategies (i.e., collective boundary and boundary turbulence management). An online survey with Facebook users (*N* = 305) finds that the three Facebook motivations are positively correlated to users’ self-extension to Facebook. The motivations for using Facebook are positively associated with the management of different layers of privacy boundaries (i.e., basic, sensitive, and highly sensitive), when Facebook self-extension is mediated. In addition, the three motives have indirect associations with potential boundary turbulence management mediated by Facebook self-extension. Extending the classic idea that privacy is deeply rooted in the self, the study demonstrates that perceiving an SNS as part of the self-system constitutes a significant underlying psychological factor that explains the linkage between motives for using SNSs and privacy management.

## Introduction

Following advances in data-mining technology and the increased popularity of social media, personal information management on social networking sites (SNSs) has become a critically important yet complex issue (Wisniewski et al., 2015). Among the various SNSs, Facebook is one of the most popular SNSs in the world (Datareportal, 2021). However, it also has various privacy-related issues manifested through such cases as accidental exposure of 6 million users’ contact information in 2011 and the Cambridge Analytica data breach in 2018 (Ivanova, 2018). Facebook is deeply connected to routines and rituals in users’ daily lives (Debatin et al., 2009), and users desire to understand privacy issues pertaining to SNS uses and express privacy concerns (Rainie, 2018).

Yet, users still share a large amount of personal information on SNSs (Osatuyi et al., 2018; Tsay-Vogel et al., 2018). A longitudinal study showed that the negative relationship between Facebook users’ privacy concerns and their disclosure of personal information on Facebook has weakened between 2010 and 2015 (Tsay-Vogel et al., 2018), despite the rise of privacy infringement cases over the period. This suggests that not all social media users are equally vulnerable to privacy risks or are equally concerned about the exposure of personal information online. Also, literature reports that online users often display discrepancies between privacy concerns and actual disclosure behaviors (Baruh et al., 2017). However, little is known regarding how different types of SNS usage shape one’s privacy management decisions and its underlying psychological mechanism.

To deepen our understanding of the factors that influence SNS users’ privacy management, the study integrates the notions that privacy is deeply rooted in the self (Altman, 1975) and that social media are often regarded as extended self by users (Belk, 2016). Grounded in those notions, this study sets out to investigate how different self-derived motivations of using Facebook influence users’ privacy management decisions. Specifically, the study examines the extent to which individual users regard their Facebook profiles as a part of the self (i.e., self-extension) as the underlying mechanism of those effects. Belk (1988) suggested that people tend to regard their important possessions as a part of their self-concept, which he labeled as “self-extension.” More recently, the notion of self-extension has been extended suggesting that one’s digital possessions (e.g., social media profile and cell phone) constitute an important part of extended selves amongst digital media users (e.g., Clayton et al., 2015; Belk, 2016).

According to Communication Privacy Management theory (CPM; Petronio, 2002), privacy management on Facebook can be viewed as managing “privacy boundaries” between the *self* (i.e., the owner of personal information) and various groups of others. Its conceptualization is based on how Altman’s (1975) viewed the privacy concept, such that privacy is about controlling others’ access to the self. We, therefore, posit that how much individual users extend themselves to one’s Facebook page, which can be regarded as a digital possession, will significantly influence their deliberate and continuous management of privacy on Facebook.

In addition, this study extends our understanding of privacy management on Facebook, by shedding light on how SNS use derived from different motives shapes users’ privacy management decisions. We expect that three major motives for using Facebook (i.e., self-expression, belonging, and archiving memory) lead users to regard Facebook as their extended selves; the expectation is that the 3 self-related motives for using Facebook will lead to self-extension to Facebook, en route to influencing individuals’ privacy management behaviors on Facebook.

All in all, this study is guided by the following overarching research question: *How do the three motivations of using a given SNS shape users’ privacy management through self-extension to the SNS?* Specifically, the study focuses on the facets of privacy boundary management, suggested by CPM: (1) the way Facebook users set their privacy boundaries that are shared with others in accordance with the sensitivity level of the information (i.e., management of collective privacy boundaries) and (2) the Facebook users’ active monitoring of potential risks of privacy breakdowns (i.e., management of potential boundary turbulence).

The study is expected to add new insights to the literature of social media, self, and online privacy research, by applying the concept of self-extension to explore privacy management behaviors in SNS contexts. Also, social media service providers will benefit from this study as its findings will enhance their understanding of SNS users’ information management strategy and psychological factors associated with it.

## Literature Review

### Motivations for Using SNSs as Antecedents to SNS Self-extension

According to the uses and gratifications (U&G) perspective, “motivations” are key forces that drive individuals to adopt different media options (Ferris and Hollenbaugh, 2018). Following this approach, this study defines SNS motivations as the key reasons that drive a user to engage in SNS activities. As SNSs have become an integral part of media use, communication scholars have attempted to answer the fundamental question of what constitutes the major motivations for using social media (e.g., Smock et al., 2011; Seidman, 2013; Jung and Sundar, 2018; Spiliotopoulos and Oakley, 2020). Research on Facebook motivations suggests that Facebook use is largely derived from self-related motivations, including self-expression, belonging, and archiving personal memories. The next section discusses the three motivations for using Facebook that are expected to contribute to Facebook self-extension, thereby shaping Facebook users’ privacy management decisions.

#### Self-Expression

Self-expression, which is considered a basic human need, enables an individual to differentiate from others, deliberate one’s own values and needs, and validate his/her self-concepts (Kim and Ko, 2007). Various technological affordances provided by SNS platforms such as text, photo, and video sharing let users fulfill self-expressive motives. Thus, numerous existing studies identify *self-expression* as one of the major motives for using Facebook (e.g., Smock et al., 2011; Seidman, 2013; van Dijck, 2013). Various tools that Facebook provides, including wall posting and profile updates, allow users to achieve self-expressive goals by sharing information about themselves with other users connected through Facebook (Seidman, 2013; Walsh et al., 2020). Moreover, conscious self-promotion or self-presentation is an important aspect of self-expression on SNSs (Ranzini and Hoek, 2017). Using Facebook, individual users are capable of engaging in one-to-many, “mass self-communication” (Castells, 2009), such that users communicate personal information to a larger audience on Facebook. Therefore, although Facebook is generally connected to real names and offline networks (Ranzini and Hoek, 2017) and often reflects the actual self (Back et al., 2010), its users tend to express themselves to their audience in their online networks (Utz et al., 2012).

#### Belonging

The *motivation for belonging* through interacting with others in a social network also constitutes a major motive for using Facebook (e.g., Smock et al., 2011; Seidman, 2013; Park and Lee, 2014; Jung and Sundar, 2018; Jung and Sundar, 2018). Network creation and communication enabled by technological features, including commenting, posting, and chatting, facilitate the fulfillment of human motivation for belonging. For example, Lin and Chu (2021) found that the need for emotional support from others made the users feel emotionally close to Facebook. Also, a recent study with older users found that Facebook use, including customization of profile pages and commenting on others’ posts, fulfilled the need for relatedness with others (Jung and Sundar, 2018).

The importance of in-group members in the development of self-perception and identity is often acknowledged by the classic philosophy and social psychology literature, suggesting that we develop our sense of self partially with others’ help (Foucault, 1978). The concept of social identity further underscores the view that self-concept is not solely individuated. Instead, our relationships with others in social groups significantly influence how we perceive ourselves (Triandis et al., 1988; Ellemers et al., 2002).

#### Memory Archiving

In addition to expressive and social motivations, “*archiving self”* is another self-related motivation for using Facebook. This motivation is expected to contribute to Facebook self-extension by functioning as “distributed memory,” a distinctive route to digital self-extension (Mittal, 2006; Belk, 2016). Although the majority of Facebook research in the communication area has described Facebook as a media platform for “social” purposes, the archival motive constitutes another important reason why individuals use Facebook (e.g., Good, 2013; Kaun and Stiernstedt, 2014; Sinn and Syn, 2014). With the new set of technological features that general social media platforms provide, people can now archive or “outsource” their memories more efficiently than ever. For instance, utilizing wall posts, photo albums, and video sharing features on Facebook, users reorganize and store important personal memories in various formats, thereby using the site as a repository or a database for personal identity and history (Sinn and Syn, 2014). Good (2013) suggests that Facebook is analogous to scrapbooks in that both are “personal media assemblages” (p. 559) that enable users to document friendships and related personal memories, though Facebook is also equipped with interactive navigation features.

### Digital Self-Extension as an Underlying Mechanism of Privacy Management on SNSs

Due to recent advances in information technologies, particularly the wide adoption of social network sites (SNSs) such as Facebook, SNSs function as important venues for creating and managing digital selves. As a result, individuals tend to view their important digital possessions, including SNSs, as an “extended self,” to varying degrees (Belk, 2016). At the same time, individuals need to manage the flow of personal information *through* various SNS platforms. Combining the rich tradition of privacy research, which links privacy and the self, and the concept of digital self-extension (Belk, 2016), this study investigates how perceiving Facebook as an extension of the self-system, derived from self-related Facebook motivations, impacts the individual’s privacy management behaviors.

The self-extension theory (Belk, 1988) suggests that “self” consists of not only our body, thoughts, ideas, and experience, but also “things that one feels attached to” (p. 141). This theory explains that an expanded view of the “self” can enhance our understanding of how our possessions contribute to our sense of existence as human beings. Given that body and mind are often considered inseparable, “knowingly or unknowingly, intentionally or unintentionally, we regard our possessions as parts of ourselves” (Belk, 1988, p. 139). In response to the rapid expansion of digital technologies, Belk (2016) has updated the concept of self-extension to incorporate the impact of digitization of possessions on the formulation of the extended self. As people increasingly extend themselves to digital possessions, including SNSs, blogs, digitized data, videos, and photos (Belk, 2016), the self constructed in the digital space has become an important part of the definition of one’s “self.” Using the self-extension concept, prior studies supported the notion that media technology cherished by its users becomes their extended self, such that they feel anxious when separated from the extended digital selves, like mobile phones (Clayton et al., 2015; Han et al., 2017).

Self-extension in the digital age is different from self-extension in the pre-digital age due to the unique characteristics of digital media technologies (Belk, 2016). Belk discusses 2 principal characteristics of digital possessions that foster a unique form of self-extension when compared to self-extension to physical possessions. In the context of digital media, many of our possessions are digitized and become invisible (i.e., *dematerialization*; e.g., photos and music), and therefore can be easily shared with others online (i.e., *sharing*). These characteristics of digital media technologies allow consumers to extend themselves to their digital possessions in the following three ways.

First, individuals easily create digital representations of the self on digital interfaces using avatars, social media profiles, photos, and videos (i.e., *re-embodiment*). Specifically, compared to anonymity-based social media platforms that allow experimentation with alternative selves, such as MUDs (Multi-User Dungeons) and chatrooms (e.g., Turkle, 1995), Facebook, one of the most popular “non-anonymous” social network sites lets users reconstruct their offline life and personal networks on an online environment. By doing so, it allows users to re-assemble and present the self largely through online interactions with their existing social connections (Wittkower, 2014). Thus, Facebook uses derived from the *self-expression* motive are particularly relevant to this aspect of digital self-extension.

Second, the self can be collaboratively constructed using digital possessions (e.g., blogs or social media pages) through interactions with others online (i.e*., co-construction of self*). On SNS platforms, a large part of self-construction is more social than personal, relying on a process that involves input from others (Belk, 2016). Facebook users tend to implicitly claim their identities by stressing identities associated with social groups through wall posts and photos depicting themselves in social contexts (e.g., Zhao et al., 2008; Turkle, 2011). Such curated and documented interactions on the Facebook interface can function as a “looking glass” that constitutes the “digital self” (Zhao, 2005) jointly created in collaboration with others in one’s social networks. Through these processes, one’s Facebook page becomes his/her extended self, built collaboratively with others in his/her social networks. Therefore, we posit that seeking a sense of *belonging* is another motivation for using Facebook that positively relates to one’s self-extension to Facebook.

Lastly, digital media, including online photo albums and blogs, enable individuals to store and archive their memories by linking their lives with other people online (i.e., *distributed memory*). According to Belk (1988), scrapbooks and photo albums are common meaningful possessions that people generally extend themselves to, as those possessions provide memory cues to people that they can associate with important events and individuals in their lives. Therefore, the study expects that *archiving personal memory* is another self-related motivation for using Facebook that contributes significantly to Facebook self-extension.

These 3 distinctive means of digital self-extension that Belk (2016) outlines (i.e., re-embodiment, co-construction of self, and distributed memory) are closely related to the major self-related motives for using Facebook identified by existing research—self-expression, belonging, and archiving memory, respectively. We thus hypothesize that:

H1: (a) *Self-expression, (b) Belonging, and (c) Archiving* motivations will be positively associated with Facebook self-extension.

### Privacy Management Behaviors on Facebook: Setting and Monitoring Privacy Boundaries

Facebook users need to make constant decisions regarding disclosure or withdrawal of private information in order to fulfill socially and personally oriented Facebook motivations. As Boyd (2010) denotes, a conversation on someone’s Facebook wall is “public by default, private through effort,” whereas an offline face-to-face conversation in a hallway is “private by default, public through effort.” The main reason for this difference is that information posted on SNSs, including Facebook, is more persistent in nature and can be more widely shared through online networks as compared to the conversation conducted in a hallway. Hence, privacy on SNSs requires more careful and consistent management than privacy in offline conversational contexts. In this context, this study explores privacy management on Facebook by following the perspective that privacy is “neither a right to secrecy nor a right to control but a right to appropriate flow of personal information” (Nissenbaum, 2009, p. 127). This notion resonates with Altman’s (1975) claim that privacy management is a dynamic process that involves “selective control of access to the self or to one’s group” (p. 18). Thus, traditional privacy research in social psychology has explicated and investigated privacy by linking it to the concept of self (Westin, 1967; Altman, 1975). Building on this foundation, we posit that if Facebook is not perceived as part of the self-system, through self-extension, users would not engage in deliberate and continuous privacy management behaviors on Facebook.

We thus argue that privacy decisions will be largely influenced by the degree to which the psychological connectedness that users feel between the self and the personal space created on Facebook. The current study attempts to understand Facebook privacy management behaviors by shedding light on the relationship between Facebook and the *self.* Specifically, we draw on the Communication Privacy Management theory (CPM; Petronio, 2002). CPM argues that privacy management should be considered as a rule-based decision-making process to manage privacy boundaries between the *self* and others to accomplish a given communication goal, which is now widely adopted to understand privacy management on SNSs (e.g., Zlatolas et al., 2015).

The basic premise of CPM is that people believe that they have rights to own private information, and therefore, have the right to protect and control others’ access to it. This sense of control helps to explain why the theory metaphorically illustrates privacy management as “*boundary*” management. When private information is not at all disclosed to others, it can be described as being kept inside of the *personal privacy boundary*, and no one else aside from the owner has a right to it. In the context of privacy management on Facebook, *privacy ownership* of private information is determined through one’s decision to “disclose” vs. “withdraw” his/her information.

However, privacy management on Facebook is not as simple as the dichotomous “disclose or withdraw” decision, because privacy management on Facebook entails controlling *collective privacy boundaries* created through sharing private information selectively with others. When private information is shared through posts, profile pages, or comments on Facebook, it transcends the personal privacy boundary and resides within a co-owned collective boundary (Child and Petronio, 2011). On Facebook, a user can determine the thickness of the boundary by choosing the breadth of co-owners (i.e., other users with access to the user’s information) of the collective privacy boundary. For highly sensitive personal information (e.g., mobile phone numbers), users can create a *thicker wall* (Petronio, 2002), by strictly limiting others’ access to it. For more basic information such as gender, they can create a *thinner wall* by sharing the information publicly. Through this process of creating multiple collective privacy boundaries, private information strata are made in accordance with the sensitivity of personal information. Using the metaphor of “multiple layers of an onion,” Jin (2013) identified multiple layers of private information disclosed on Twitter. The study showed that Twitter users disclose less private and sensitive information (i.e., the outer layer of the private disclosure onion), such as daily activities and entertainment most easily, while hardly revealing health-related private information (i.e., the inner layer of the private disclosure onion). Similarly, Chang and Heo (2014) found that different motives for using Facebook are associated with distinct choices for disclosing various levels of private information. Collectively, making decisions on where to place multiple levels of collective privacy boundaries in a given context, thereby creating multiple strata of privacy boundaries, is an integral part of privacy management.

In addition, privacy management on Facebook includes managing potential risks of “*boundary turbulence*.” Creating collective privacy boundaries by sharing private information with others increases the risk of “boundary turbulence,” which refers to the disruption of privacy boundary management when personal information leaks to other parties without the owner’s control or permission (Petronio, 2002). Privacy turbulence can occur more easily in SNS environments than elsewhere since individuals in SNS contexts engage with various social networks that provide different technological features allowing the easy and immediate transference of information. In the context of Facebook, for example, users can regularly check the privacy settings of previous posts when they review their Facebook walls or check who can see their friends’ posts in which they themselves are tagged. Thus, even after setting collective privacy boundaries, active and consistent monitoring of the boundaries is required for effective management of privacy on SNSs in order to avoid privacy turbulence. Hence, monitoring and managing privacy boundaries to minimize the potential risk of privacy turbulence is examined as one of the key dimensions of privacy management on SNSs.

All in all, combining the self-extension concept and CPM, our study is grounded in the following key propositions: (1) Facebook can be regarded as one’s extended self, which is derived from self-related Facebook motivations, and (2) privacy management is about controlling the boundaries between the *self* and others. Therefore, (3) the degree to which one feels Facebook as her/his extended self will be positively associated with privacy management strategies. We expect that self-extension derived from self-expression, belonging, and archiving motives will be positively associated with privacy disclosure in terms of both width of boundaries and the amount of information shared within the boundaries (i.e., collective boundary management). But at the same time, self-extension will be positively related to careful monitoring and management of the boundaries to avoid any unexpected information leakage (e.g., boundary turbulence management).

Hence, we hypothesize that:

H2: For Facebook users, 3 Facebook motivations (self-expression, belonging, and archiving memory) will have positive indirect associations with (a) collective privacy boundaries (i.e., the amount of information disclosed and the breadth of co-owners of the information in each private information stratum), and (b) potential boundary turbulence (i.e., active monitoring of collective boundaries on Facebook), when mediated by Facebook self-extension.

## Method

### Procedure and Participants

An online survey was conducted with Facebook users aged 18 and older residing in the United States. The participants were recruited from Qualtrics online panel in March 2017 for 2 days. Qualtrics has randomly selected people who met the recruitment criteria from their online panel pool. In total, 305 participants completed the survey. Participants (*N* = 305; *Mean*_*age*_ = 46.15, *SD*_*age*_ = 15.36) consisted of 221 females (72.5%) and 84 males (27.5%). Q-Q plots did not indicate any significant outliers in the dataset; therefore, no participants were removed from the data. The survey questionnaire was reviewed by the ethics review board of the first author’s institute. Upon the completion of the study, Qualtrics compensated survey respondents with credit points, in line with consumer research industry standards.

A recent report from Pew Research shows that Facebook is used by the majority of social media users in the United States. across a broad range of age groups (70% of 18–29 years old, 77% of 30–49 years old, 73% of 50–64, 50% of 65, and older; Auxier and Anderson, 2021). As for gender, US Facebook users consist of 55% female and 45% male (Statista, 2021). Our sample overrepresents older Facebook users and female users. The effects of age and gender were statistically controlled in our analyses as reported in the result section. The majority of the participants identified themselves as Caucasian (83.6%) followed by African American (5.9%) and Asian (5.9%). Most of them were active Facebook users, such that about 6 out of 10 reported using Facebook more than once a day.

Table 1 presents the demographic characteristics and Facebook usage of the participants.

**TABLE 1:** Sample characteristics (*N* = 305).

		*n* (Frequency)	%
**Gender**			
	Female	221	72.5%
	Male	84	27.5%
**Ethnicity**			
	Caucasian	255	83.6%
	African American	18	5.9%
	Asian/Pacific Islander	18	5.9%
	Hispanic/Latino	10	3.3%
	Native American/American Indian	2	0.7%
	Other	2	0.7%
**Education**			
	Did not complete high school	8	2.6%
	High School/GED	80	26.2%
	Some College	119	39.0%
	Bachelor’s Degree	74	24.3%
	Master’s Degree	19	6.2%
	Advanced graduate work or Ph.D	5	1.6%
**Facebook Use (About how often do you use Facebook?)**			
	Once a week	15	4.9%
	A few times a week	45	14.8%
	Once a day	50	16.4%
	More than once a day	195	63.9%
**# of Friends on Facebook (About how many Facebook friends do you have?)**			
	0–50	67	22%
	51–100	67	22%
	101–250	76	24.9%
	251–500	58	19%
	501–1000	25	8.2%
	1001 or more	12	3.9%

### Measures

*Facebook motivations* were assessed by asking participants to indicate their agreement with statements that describe Facebook usage derived from different motives, using 5-point Likert scales (1 = strongly disagree; 5 = strongly agree). The *self-expression motive* was measured with 3 items from Smock et al. (2011), developed based on Papacharissi and Mendelson (2011); e.g., “to tell others about myself” (*M* = 3.63 *SD* = 0.89; α = 0.78). Five items for *the sense of belonging* motive were adopted from Seidman (2013); e.g., “to feel included” (*M* = 3.46, *SD* = 0.84; α = 0.83). Although Seidman (2013) operationalized the original belonging motive as a construct consisting of 2 dimensions (i.e., acceptance seeking and connection/caring), we combined those 2 dimensions into a unidimensional construct due to the conceptual similarity between the 2 dimensions. An exploratory factor analysis (EFA) using the Principal Axis Factoring with Oblimin Rotation method has been implemented. The results revealed that only one factor showed an eigenvalue greater than 1 (eigenvalue = 2.99), suggesting that the 5 items measure a unidimensional construct. The factor loadings of all 5 items were over.60 (“to feel included”: 0.60; “to make others feel closer to me”: 0.71; “to feel closer to others”: 0.83; “to show caring for others”: 0.72; “to support others”: 0.67). In addition, the Kaiser–Meyer–Olkin sampling adequacy measure was 0.81, above the recommended value of 0.60, and Bartlett’s test of sphericity was significant, χ^2^ (10) = 576.47, *p* < 0.001.

Lastly, the motive for *archiving* personal memories was measured with 7 items adapted from Sinn and Syn (2014); e.g., “Facebook allows me to access my daily memories easily.” (*M* = 3.55, *SD* = 0.86, α = 0.90). Sinn and Syn had 6 items, but we separated one of the items, “Facebook allows me to access my everyday stories easily and frequently” into 2 items: “Facebook allows me to access my everyday stories easily” and “Facebook allows me to access my everyday stories frequently.”

*Self-extension to Facebook* was measured with 8 items on 5-point Likert scales (1 = strongly disagree; 5 = strongly agree). The measurement items were drawn from Sprott et al. (2009); e.g., “I consider my Facebook profile to be a part of myself” (*M* = 3.12, *SD* = 1.04, α = 0.95). The composite scale of this construct was created by averaging the 8-item scores.

*Collective boundary management* was measured by asking participants to indicate how each of the 24 personal information items was set to be shown to different groups of people: not entered or only me = 0; some Facebook friends = 1; all Facebook friends = 2; and public = 3. Then, following Chang and Heo (2014), we divided the 24 personal information items into 3 categories based on the level of sensitivity of the information; basic (9 items; e.g., college, current city, hometown, and language), sensitive (8 items; e.g., current workplace, past workplaces, email, birth date, and birth year), and highly sensitive (7 items; e.g., mobile phone number, interested in men or women, and religious views). The sum of the disclosure scores in each sensitivity category (basic, moderately sensitive, and highly sensitive) was divided by the number of information items for each category (e.g., Basic information disclosure = the sum of basic information disclosure scores/9). This score has been used as the Information Disclosure Index (IDI); the more information items disclosed to a wider group of people, the higher IDI was. While Chang and Heo (2014) assessed users’ self-disclosure by counting the number of information provided in each category (disclose vs. not disclose), our measure gauged the *width* of collective privacy boundaries in addition to the number of personal information items disclosed. IDI was highest for the basic personal information (*M* = 1.24, *SD* = 0.76) followed by the moderately sensitive information (*M* = 0.95, *SD* = 0.70), and the lowest for the highly sensitive information (*M* = 0.87, *SD* = 0.73). The paired sample *T*-test results showed that the IDIs of 3 strata are significantly different from each other [basic – moderately sensitive: *t* (304) = 9.35, *p* < 0.001; basic - highly sensitive: *t* (304) = 11.887, *p* < 0.001; moderately sensitive - highly sensitive: *t* (304) = 2.81, *p* < 0.001].

*Boundary turbulence management* was assessed with 3 items developed by the authors. On Facebook, there are 3 key facets where personal information can be shared and managed: (a) by sharing posts on the wall, (b) sharing personal information on the profile, and (c) tagged posts shared by others. With 3 items, we have measured the extent to which the users actively and regularly check how their information is shared through these 3 facets. Therefore, the items were designed to assess Facebook users’ general behaviors to regularly monitor and maintain with whom their personal information is shared on their wall posts, profile page, posts with tags. These items were measured on 5-point Likert scales (1 = strongly disagree; 5 = strongly agree). The composite scale of this construct was created by averaging the 3 item scores (*M* = 3.43, *SD* = 0.95; α = 0.73).

*Control variables* include individual difference items that are known to influence online privacy management behaviors. Studies showed that individuals tend to show different patterns of privacy disclosure and management online in accordance with demographic factors, including age, gender, and education (Kisekka et al., 2013; Choi and Kim, 2014; Baruh et al., 2017). Also, general self-efficacy and aptness of using communication technology (Sundar and Marathe, 2010; Kisekka et al., 2013; Kang and Shin, 2016) have been found to influence internet users’ privacy-related perceptions and behaviors. Twelve items adapted from Sundar and Marathe (2010) were used to measure general self-efficacy and aptness of using communication technology; e.g., “Using any technological device comes easy to me” (*M* = 3.5, *SD* = 0.75, α = 0.89). In addition, the frequency of Facebook use and the network size (Choi and Kim, 2014; Lankton et al., 2017) have been found to influence privacy-related attitudes and management behavior among Facebook users. All in all, demographic factors (i.e., age, gender, and education), power usage, frequency of Facebook use, and the number of Facebook friends were controlled to isolate the effects of these individual differences from the relationships among Facebook motives, Facebook self-extension, and privacy management.

See Supplementary Appendix A and B for the measures and partial correlations (controlling for the control variables listed above) between the variables.

## Results

To test the hypotheses, we performed a path model analysis using AMOS (version 26) statistical program. Through this path model analysis, we estimated the direct paths between Facebook motivations (independent variables) and self-extension (mediator), as well as the direct paths between self-extension (mediator) and boundary management strategies (dependent variables). The analysis also estimated the indirect effects of Facebook motivations on privacy management behaviors mediated by Facebook self-extension employing 2,000 bootstrapped samples. With VIF of predictors between 1.04 and 3.51, no multicollinearity issue among the predictors was detected. Kolmogorov-Smirnov was performed with residuals to test the normality of residuals, showing that residuals were normally distributed, *D* (305) = 0.05, *p* = 0.09.

**FIGURE 1:**
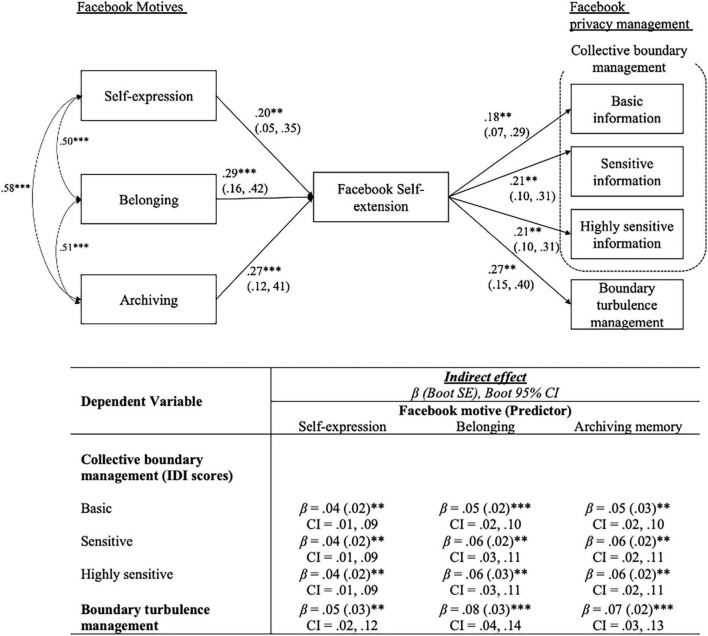
Path analysis results. Goodness of Fit: *x*^2^ = 14.8, *df* = 12; CFI = 0.998, TLI = 0.987, AGFI = 0.940, RMSEA = 0.028 (90% CI:0.00, 0.07); Path entries are standardized coefficient (*β*) and 95% CI; *p < 0.05; **p < 0.01; ***p < 0.001; gender, age, education, poweruse, # of friends, frequency of usage were included in the model as control variables.

The path model produced a good fit χ2 = 14.8 (*df* = 12, *p* < 0.001), RMSEA = 0.028 (90% Confidence Interval [CI]: [0.00, 0.07]), CFI = 0.998, TLI = 0.987, and AGFI = 0.940 (Figure 1). As hypothesized in H1, self-expression, belonging, and archiving motives were found to be positively associated with Facebook self-extension. Thus, H1a, H1b, and H1c were supported. Regarding the indirect effects (H2), the results revealed that all 3 Facebook motivations were found to be indirectly associated with IDI of basic, sensitive, and highly sensitive information when Facebook self-extension mediated. In addition, when Facebook self-extension mediated, all three motives had significant indirect effects on boundary turbulence management. Therefore, H2a and H2b were supported. Figure 1 summarizes the path model analysis results.

Regarding the effects of control variables, the results indicated that male Facebook users, compared to female users, were more likely to extend themselves to Facebook, reveal sensitive information to broader groups, and carefully manage privacy boundaries on Facebook. The size of the network also had a positive association with Facebook self-extension and collective boundary management. The frequency of Facebook was positively correlated only with the management of basic information. The aptness level of using media technology (i.e., power usage) had a positive association with boundary management. Table 2 summarizes the associations between control variables and endogenous variables.

**TABLE 2:** Effects of control variables on mediator and dependent variables.

Control variables	Facebook self-extension	Collective boundary management			Boundary turbulence management
		*Basic*	*Sensitive*	*Highly sensitive*	
Gender (1 = male; 2 = female)	−0.15**	–0.10	−0.16**	−0.14*	0.15**
Age	0.08	–0.10	–0.11	−0.14*	–0.08
Education	–0.06	–0.05	–0.09	–0.08	–0.06
Power usage	–0.05	0.04	–0.04	–0.00	0.25**
Frequency of using Facebook	0.07	0.12*	0.08	0.10	–0.05
Network size on Facebook	0.10*	0.24**	0.15**	0.15*	0.06

entries are standardized coefficient (β); *p < 0.05, **p < 0.01.

## Discussion

### Theoretical Implications

One of the key theoretical contributions of the study is that we investigated SNS users’ privacy management with an assumption that SNS information management is multifaceted, often going beyond the “disclosure-withdrawal” dichotomy (Christofides et al., 2009). In fact, many SNS platforms provide sophisticated information management tools that enable users to specify the types and levels of information disclosure. In addition, although numerous previous studies have focused on the disclosure aspect of privacy management behaviors on SNSs (e.g., Taddicken, 2014; Cheung et al., 2015; Choi and Bazarova, 2015; Chen et al., 2016), a growing number of studies have also suggested that SNS users employ various strategies to control and manage the flow of their personal information on social media (e.g., Child and Agyeman-Budu, 2010; Chen and Chen, 2015; Child and Starcher, 2016). Thus, it is important to employ a more granulated approach to theorizing privacy management behaviors on SNSs, as well as the underlying mechanisms that shape the multifaceted aspects of privacy management on SNSs.

Derived from CPM (Petronio, 2002), our study addressed the gap in the existing research literature by examining multiple aspects of privacy boundary management on SNSs—collective privacy boundaries and potential boundary turbulence. Thus, our view on privacy management goes beyond one’s decisions regarding the level of personal information disclosure. Instead, it embraces the active and continuous management of personal and collective privacy boundaries, through both strategic disclosures and monitoring, which reflect how current SNS users actually manage privacy.

The study found that 3 Facebook usage motivations (self-expression, belonging, and archiving personal memory) had indirect associations with privacy management behaviors, mediated through Facebook self-extension. Thus, the result supports the study’s core proposition that self-extension to Facebook is a significant psychological mechanism underlying how different self-related motives shape one’s privacy management strategy on Facebook. The previous research findings that SNS users’ privacy-related decisions can be derived from social approval and validation motives (Choi and Bazarova, 2015; Dienlin and Metzger, 2016; Farci et al., 2017). In addition to the social (belonging) motive, the study results also support the commonsensical notion that information disclosure is a significant means for fulfilling self-expression and memory archiving motives for using SNSs. Yet, a noteworthy implication of our study lies in the indirect effect results, as these associations were significant when the Facebook motives led users to perceive Facebook as an extended self. That is, belonging, self-expression, and archiving motives for using SNSs can influence information disclosure behaviors only through an intervening psychological step, where a given SNS platform is incorporated into one’s self-system. This finding corroborates the traditional conceptualizations of privacy management as an act to achieve personal goals of self-realization (Westin, 1967) and to create self-identity (Altman, 1975). Even though the media environment has dramatically changed over the past decades, the importance of the “self” and motives derived from the self remains the same when it comes to managing privacy on digital media. Our results also highlight the roles played by both social and non-social motives of media usage in understanding social media users’ privacy management strategies, adding important knowledge to the CPM literature.

In addition, we have developed the information disclosure index (IDI) to assess collective boundary management. The index formula was designed to gauge the degree of the width of collective privacy boundaries as well as the number of personal information items disclosed. It is because collective privacy boundary management concerns selective disclosure of personal information, which conceptually aligns with the definition of collective boundary management. We thus believe that our development of the measurement constitutes a unique contribution to this study.

### Practical Implications

Our study provides valuable practical implications for SNS providers and users. First, the study results demonstrate that Facebook users who are motivated to express themselves, feel included, or to curate and store personal memories using Facebook are more likely to regularly monitor whether their personal information is shared with unwanted audiences. The Cambridge Analytical data breach, along with other scandals related to infringements of SNS users’ personal information, has heightened people’s concerns about privacy on social media (Rainie, 2018). It is thus critical that SNS service providers respond to this public concern in order to retain their users and provide them with a positive SNS experience.

Following the findings of this study, we suggest that SNS services provide various tools that guide users and remind them to check and modify their privacy settings when they engage in self-expressive activities (e.g., posting photos), relationship building (e.g., interacting on Facebook communities) and personal archiving related (e.g., creating photo albums on Facebook) activities. Moreover, social media can provide more detailed options for monitoring and managing their privacy boundaries. For example, Facebook allows users to check how their profiles are viewed by the “public,” by clicking the “View as” button on the profile page. However, the “View as” feature only provides the “public” option. Given that users are increasingly motivated to carefully manage their information on Facebook, especially when they extend themselves to it, Facebook can provide more options (e.g., view as “friends” or specific group/person) to facilitate their detailed privacy management.

Second, the results also showed that Facebook users are more likely to view Facebook as part of their self-systems when they are motivated to express themselves, seek a sense of belonging, and archive personal memories through Facebook. The literature on self-extension suggests that self-extension to one’s possessions causes individuals to feel both cognitively and emotionally attached to those items (e.g., Kiesler and Kiesler, 2005; Clayton et al., 2015; Han et al., 2017). Based on our result, offering platforms where users can efficiently express themselves, receive support from each other, and store their memories will appeal to Facebook users. For example, active integration of AI-based AR stickers and filters will encourage the users to fulfill the self-expressive motives; the algorithm-based notification system that alerts friends’ activities and postings of users’ interests will satisfy their needs for belonging. In addition, providing algorithm-based archiving features (e.g., automated posting or photo categorization) will also help the users easily fulfill their archiving motives.

The study also reveals that extending the self to Facebook through the 3 self-related motives can lead users to share personal information on Facebook. Although the results also showed that Facebook self-extension promotes active monitoring of information flows online, it is almost impossible for users to perfectly control the flow of information once that information is already shared with others on social media. Thus, the awareness of privacy risks associated with social media usage should be continuously communicated to users, especially with those who are more likely to regard Facebook as a part of themselves. It is also important that SNSs provide features that prompt users to review and confirm who can access their shared information especially among those who are regarded as being highly attached to the site.

### Limitations and Future Directions

The limited scope of the current study provides some valuable guidance for future research. First, this study examined a single social media platform—Facebook. We focused on Facebook as it is the most popular social media platform worldwide and has also generated substantial privacy concerns in recent years. However, as different social media platforms appeal to different groups of users, fulfilling different types of psychological needs, findings from this study may not be applicable to other social media contexts. For example, people tend to use Pinterest to discover new trends and get inspired. Instagram is used to showcase individuals’ lives in unique and sometimes artful ways. LinkedIn is used primarily as a business network. Future research could examine various types of social media to address how users shape and extend the self through different social media platforms and whether the choice of networks affects their information management decisions.

Second, although this study examined various types of Facebook usage motives (self-expression, belonging, and archiving memories) that are closely related to 3 dimensions of digital self-extension (re-embodiment, co-construction of self, and distributed memory), it did not examine other important motivations for engaging in social media, including entertainment, information seeking, convenience, altruism, and self-status seeking (e.g., Lin et al., 2017). Future research will generate new breakthroughs by examining a wider range of motives that are possibly associated with users’ self-extension to social media platforms, as well as their information management behaviors.

Third, our data overrepresent female and older Facebook users although their effects were statistically controlled in our data analyses. In addition, we tested our hypotheses using cross-sectional data. To confirm the causal relationship between variables tested in the study, future studies might conduct experimental analyses, by manipulating self-extension to the social media platform. As one example, customization of the interface could be a good way to manipulate the level of self-extension.

Finally, we created the measurement for the privacy boundary turbulence management as we could not find a suitable instrument capturing the continuous monitoring and controlling of privacy boundaries in the Facebook context. However, this study did not carry out a thorough validation test of the new instrument. Given that continuous monitoring and management of privacy boundaries have become a critical privacy management behavior in the social media context, future research should develop and validate the measurement for this construct.

## Conclusion

Social media have become important components of our self-construction. We actively form our virtual selves by expressing ourselves, sharing various information with others, and curating and archiving our memories on our social networking sites. This study investigates how these self-related motivations for using Facebook are associated with different privacy management strategies through self-extension to this social media platform. Drawing upon the CPM approach, our study investigated two particular aspects of privacy boundary management, in recognition of the fact that information management on SNSs often goes beyond the “disclosure-withdrawal” dichotomy. Supporting the classic notion that privacy represents the selective control of information flows about the self, our study showed that regarding an SNS as an extended self, derived from self-related motives for using the SNS platform, is significantly linked to the management of one’s own privacy boundaries on the platform. Findings from this study will enhance our understanding of how users perceive social media platforms in relation to their self-perception and how they manage privacy as a result, thereby providing meaningful implications for social media practitioners as well as users.

## Data Availability Statement

The raw data supporting the conclusions of this article will be made available by the authors, without undue reservation.

## Ethics Statement

The studies involving human participants were reviewed and approved by Nanyang Technological University. The patients/participants provided their written informed consent to participate in this study.

## Author Contributions

HK generated research questions, performed literature review, managed data collection and analysis, and wrote up the manuscript. WS generated research questions, helped in data collection and analysis, and wrote up the manuscript. Both authors contributed to the article and approved the submitted version.

## Conflict of Interest

The authors declare that the research was conducted in the absence of any commercial or financial relationships that could be construed as a potential conflict of interest.

## Publisher’s Note

All claims expressed in this article are solely those of the authors and do not necessarily represent those of their affiliated organizations, or those of the publisher, the editors and the reviewers. Any product that may be evaluated in this article, or claim that may be made by its manufacturer, is not guaranteed or endorsed by the publisher.
