# Supplementary material for: When Facebook Becomes a Part of the Self: How Do Motives for Using Facebook Influence Privacy Management?
Source: Front Psychol. 2021 Dec 16;12:769075. doi: 10.3389/fpsyg.2021.769075 (PMC8716453; doi:10.3389/fpsyg.2021.769075)
Supplement: Supplementary file 1 [file Table_1.pdf]

**Appendix A.** Measurement Items

| Measures | M (SD) | α | Skewness | Kurtosis |
|---|---|---|---|---|
| **Motivations of using Facebook (**1= strongly disagree; 5 = strongly agree) | | | | |
| **Self-expression** ***"I use Facebook…"*** | 3.63 (.89) | .78 | -.94 | 1.14 |
| 1. to post information about a special interest of mine.<br>2. to share information that may be of use or interest to others.<br>3. to tell others about myself. | | | | |
| **Belonging** ***"I use Facebook…"*** | 3.46 (.84) | .83 | -.73 | .90 |
| 1. to feel included.<br>2. to make others feel closer to me.<br>3. to feel closer to others.<br>4. to show caring for others.<br>5. to support others. | | | | |
| **Archiving** | 3.55 (.86) | .90 | -.85 | 1.02 |
| 1. I think of Facebook to post when I find something interesting.<br>2. I sometimes take photographs with Facebook in mind.<br>3. When I post photographs, videos, links, etc., on Facebook, I provide descriptions for them (such as when, where, what, why, who).<br>4. I look at Facebook posts to recall details about places, people, past events, etc<br>5. Facebook allows me to access my daily memories easily.<br>6. Facebook allows me to access my daily memories frequently,<br>7. Facebook is a tool I can easily use to document my personal stories. | | | | |
| **Facebook Self-extension (**1= strongly disagree; 5 = strongly agree) | 3.12 (1.04) | .95 | -.24 | -.58 |
| 1. I have a special bond with my Facebook profile.<br>2. I consider my Facebook profile to be a part of myself.<br>3. I often feel a personal connection between my Facebook profile and me.<br>4. A part of me is defined by my Facebook profile.<br>5. I feel as if I have a close personal connection with my Facebook profile.<br>6. I can identify with my Facebook profile.<br>7. There are links between my Facebook profile and how I view myself.<br>8. My Facebook profile is an important indicator of who I am. | | | | |
| **Collective Boundary Management**<br>Information items were devided into three categories based on the level of sensitivity of the information (Chang & Heo, | | | | |

| | | | | |
|---|---|---|---|---|
| 2014) as follow. Then, the sum of the disclosure scores in each sensitivity was divided by the number of information items for each category for Information Disclosure Index (IDI) | | | | |
| • **Basic (9 items)** College, High School, Current city, Hometown, Other places lived, Language, Some details about yourself, Other names, Favorite quotations | 1.24 (.76) | na | .18 | -.70 |
| • **Sensitive (8 items)** Current workplace, Past workplaces, Professional skills, Email, Birth Date, Birth Year, Website, Social link | .95 (.70) | na | .61 | -.11 |
| • **Highly sensitive (7 items)** Mobile phone, Address, Interested in men or women, Your religious views, Your political views, Current relationship status, Family members | .87 (.73) | na | .82 | -.16 |
| **Boundary turbulence management** (1= strongly disagree; 5 = strongly agree)<br>1. When I review my Facebook wall, I check privacy settings of individual posts.<br>2. When I was tagged on a post, I always check who can see that post.<br>3. I regularly check what strangers can see on my Facebook profile. | 3.43 (.95) | .73 | -.28 | -.41 |
| **Power usage** (1= strongly disagree; 5 = strongly agree)<br>1. I think most of the technological gadgets are complicated to use.*<br>2. I make good use of most of the features available in any technological device.<br>3. I have to have the latest available upgrades of the technological devices that I use.<br>4. My use of information technology has almost replaced my use of paper.<br>5. I love exploring all the features that any technological gadget has to offer.<br>6. I often find myself using many technological devices simultaneously.<br>7. I prefer to ask friends how to use any new technological gadget instead of trying to figure it out myself.*<br>8. Using any technological device comes easy to me.<br>9. I feel like information technology is a part of my daily life.<br>10. Using information technology gives me greater control over my work environment.<br>11. Using information technology makes it easier for me to do my work.<br>12. I would feel lost without information technology. | 3.50 (.75) | .89 | -.41 | .25 |
