# Supplementary material for: When Facebook Becomes a Part of the Self: How Do Motives for Using Facebook Influence Privacy Management?
Source: Front Psychol. 2021 Dec 16;12:769075. doi: 10.3389/fpsyg.2021.769075 (PMC8716453; doi:10.3389/fpsyg.2021.769075)
Supplement: Supplementary file 2 [file Table_2.pdf]

Appendix B. Partial Correlation matrix

	1. self-expression motive	2. belonging motive	3. archiving motive	4. self-extension	5. basic IDI	6. sensitive IDI	7. highly sensitive IDI	9. boundary turbulence control
1	1							
2	.61***	1						
3	.67***	.64***	1					
4	.52***	.56***	.55***	1				
5	.22***	.13*	.20***	.18**	1			
6	.19***	.15**	.19**	.20***	.71***	1		
7	.17**	.14*	.14*	.20***	.68***	.68***	1	
8	.17**	.22***	.30***	.27***	-.16**	-.07	-.10	1

Note: gender, age, education, poweruse, # of friends, frequency of usage were controlled.;
* p < .05 ; ** p < .01 ; *** p < .001
